# Comparative Gene Expression Analysis of the Human Periodontal Ligament in Deciduous and Permanent Teeth

**DOI:** 10.1371/journal.pone.0061231

**Published:** 2013-04-08

**Authors:** Je Seon Song, Dong Hwan Hwang, Seong-Oh Kim, Mijeong Jeon, Byung-Jai Choi, Han-Sung Jung, Seok Jun Moon, Wonse Park, Hyung-Jun Choi

**Affiliations:** 1 Department of Pediatric Dentistry, College of Dentistry, Yonsei University, Seoul, Korea; 2 Oral Science Research Center, College of Dentistry, Yonsei University, Seoul, Korea; 3 Division in Anatomy & Developmental Biology, Department of Oral Biology, College of Dentistry, Yonsei University, Seoul, Korea; 4 Division in Pharmacology, Department of Oral Biology, College of Dentistry, Yonsei University, Seoul, Korea; 5 Department of General Dentistry, College of Dentistry, Yonsei University, Seoul, Korea; Naval Research Laboratory, United States of America

## Abstract

There are histological and functional differences between human deciduous and permanent periodontal ligament (PDL) tissues. The aim of this study was to determine the differences between these two types of tissue at the molecular level by comparing their gene expression patterns. PDL samples were obtained from permanent premolars (n = 38) and anterior deciduous teeth (n = 31) extracted from 40 healthy persons. Comparative cDNA microarray analysis revealed several differences in gene expression between the deciduous and permanent PDL tissues. These findings were verified by qRT-PCR (quantitative reverse-transcription–polymerase chain reaction) analysis, and the areas where genes are expressed were revealed by immunohistochemical staining. The expressions of 21 genes were up-regulated in deciduous relative to PDL tissues, and those of 30 genes were up-regulated in permanent relative to deciduous PDL tissues. The genes that were up-regulated in deciduous PDL tissues were those involved in the formation of the extracellular matrix (LAMC2, LAMB3, and COMP), tissue development (IGF2BP, MAB21L2, and PAX3), and inflammatory or immune reactions leading to tissue degradation (IL1A, CCL21, and CCL18). The up-regulated genes in permanent PDL tissues were related to tissue degradation (IL6 and ADAMTS18), myocontraction (PDE3B, CASQ2, and MYH10), and neurological responses (FOS, NCAM2, SYT1, SLC22A3, DOCK3, LRRTM1, LRRTM3, PRSS12, and ARPP21). The analysis of differential gene expressions between deciduous and permanent PDL tissues aids our understanding of histological and functional differences between them at the molecular level.

## Introduction

The periodontal ligament (PDL) tissues are components of the dental apparatus that connect the tooth to the alveolar jaw bone in the area surrounding the root surfaces. Similar to tendons, collagen type I is a predominant component of PDL tissue, but collagen types III, V, VI, and XII, and proteoglycans, which are known to regulate collagen fibril formation, are also found in this tissue [1], [2]. The PDL tissues comprise various cells such as PDL fibroblasts, epithelial cell rests of Malassez, osteoblasts, cementoblasts, vascular cells, and sensory nerve cells.

There are many anatomical, embryological, functional, and structural differences between human deciduous and permanent teeth. For example, deciduous and permanent teeth exhibit different responses to external stimuli, and the sensory nerve endings are fewer and looser in permanent than in deciduous teeth [3], [4]. Because of the different responses of dental pulp tissues, the modalities of pulp therapy should differ between deciduous and permanent teeth [5]. Besides, in vitro and in vivo experiments have shown that cells originating from deciduous teeth behave differently to those from permanent teeth [6], [7].

Since deciduous teeth are resorbed and exfoliated in association with the eruption of successive permanent teeth, the periodontal tissues of the former are more easily resorbed than those of the latter [8], [9]. To explain this at the molecular level, some investigators reported that the periodontal tissues of deciduous teeth contain more bone sialoprotein and osteopontin with the Arg-Gly-Asp (RGD) sequence, to which odontoclasts bind [10], [11]. Others reported that PDL cells obtained from areas being resorbed in deciduous teeth express more of the receptor activator of the nuclear factor κ-light-chain-enhancer of activated B cells ligand (RANKL) gene, which is known to be associated with osteoclastogenesis [12], [13]. In addition, extracellular-matrix-degrading enzymes such as collagenase [14], metalloproteinases [15], [16], and mucopolysaccharidase [17] were found to be up-regulated upon the resorption of deciduous periodontium. However, these findings are not sufficient to explain the differences in the normal-functioning periodontium of deciduous and permanent teeth.

The recent development of microarray analysis allows evaluation of the expressions of large numbers of genes simultaneously, and has been used to investigate periodontal tissues [18] and periodontal cell cultures [19], [20]. Given the anatomical and functional differences between the periodontal tissues of deciduous and permanent teeth, it is reasonable to assume that there are also differences in the gene expression patterns of the cells within those tissues. Therefore, the aims of the present study were to identify and compare the gene expression patterns of human deciduous and permanent periodontal tissues in order to enhance our understanding of the molecular basis of the observed functional differences between these two tissue types.

## Materials and Methods

### PDL samples

The experimental protocol was approved by the Institutional Review Board of the Yonsei University Dental Hospital, and informed consent to participate was obtained from all of the subjects and their parents (#2-2011-0009). PDL samples were obtained from healthy permanent premolars (*n* = 38; from 4 males and 10 females, aged 10–19 years) extracted for orthodontic reasons and from anterior deciduous teeth (*n* = 31; from 14 males and 12 females aged 5–13 years) extracted for space management in 40 healthy persons. For RNA isolation, each of the extracted teeth (36 permanent teeth and 29 deciduous teeth) was immediately frozen and stored in liquid nitrogen. All of the teeth were subsequently thawed at room temperature, and the PDL tissues were obtained carefully using sterile curettes from the middle-third in permanent teeth or from the nonresorbed root surface in deciduous teeth. We mixed the PDL tissues and divided them into three equal groups, and then immediately submerged them in a RNA stabilizing reagent (RNAlater, Qiagen, Valencia, CA, USA). The remaining four teeth (two permanent teeth and two deciduous teeth) were used for immunohistochemical (IHC) staining.

### RNA isolation

The PDL tissues were homogenized using a homogenizer (Bullet Blender, Next Advance, NY, USA). The RNeasy Fibrous Mini kit (Qiagen) was then used to extract total RNA from the PDL tissues, according to the manufacturer's instructions. The extracted RNA was eluted in 25 µl of RNase-free sterile water (provided with the kit). The quality and concentration of RNA were determined by measuring the absorbance at a wavelength of 260 nm with the aid of a spectrophotometer (NanoDrop ND-1000, Thermo Scientific Inc, Rockford, IL, USA). The RNA samples used in this study had 260/280 nm ratios of ≥1.8. Half of isolated RNA was used for complementary DNA (cDNA) microarray experiments, and the other half was used for gene expression analysis with the quantitative reverse-transcription–polymerase chain reaction (qRT-PCR).

### cDNA microarray

Global gene expression analyses were conducted using oligonucleotide arrays (GeneChip Human Gene 1.0 ST, Affymetrix, Santa Clara, CA, USA). The samples were prepared according to the instructions and recommendations provided by the manufacturer. The quality and quantity of RNA were assessed using a bioanalyzer (model 2100 with RNA 6000 Nano Chips, Agilent Technologies, Amstelveen, The Netherlands).

As recommended by the manufacturer's protocol, 300-ng samples were used. In brief, 300 ng of total RNA from each sample was converted to double-stranded cDNA. Amplified RNA (cRNA) was generated from the double-stranded cDNA template using a random hexamer incorporating a T7 promoter, via in-vitro transcription, and purified with the Affymetrix sample cleanup module. cDNA was regenerated through a random-primed reverse transcription (RT) using a dNTP mix containing dUTP. The cDNA was then fragmented by UDG and APE1 restriction endonucleases and end-labeled by a terminal transferase reaction incorporating a biotinylated dideoxynucleotide. The fragmented end-labeled cDNA was hybridized to the GeneChip Human Gene 1.0 ST arrays for 16 hours at 45°C and 60 rpm, as per the GeneChip Whole Transcript Sense Target Labeling Assay Manual (Affymetrix). The chips were then stained and washed in a GeneChip Fluidics Station 450 (Affymetrix) and scanned using a GeneChip Array scanner (3000 G7, Affymetrix). The image data were extracted using Affymetrix Command Console software (version 1.1, Affymetrix). The raw file generated using this procedure provided the expression intensity data that was used for the next step.

### Analysis of microarray data

The generated expression data were normalized using the Robust MultiAverage (RMA) algorithm in the Affymetrix Expression Console software. Whether genes were differentially expressed between the three groups was determined by subjecting the RMA expression data to one-way ANOVA. Multiple testing correction was applied to the *p* values of the F-statistics to adjust for the false discovery rate. Genes with adjusted F-statistic *p* values of <0.05 were extracted. Strongly expressed genes in each test group that were up-regulated by over twofold compared to the signal value were selected for further study. The coexpression gene group, with similar expression patterns, was classified using hierarchical clustering and ***k***-means clustering with Multi Experiment Viewer software (version 4.4, www.tm4.org, Dana-Farber Cancer Institute, Boston, MA, USA). The Web-based tool Database for Annotation, Visualization, and Integrated Discovery was used to interpret the biological implications of the differentially expressed genes. These genes were then classified based on the gene function information provided in the Gene Ontology (GO), Kyoto Encyclopedia of Genes and Genomes Pathway database (http://david.abcc.ncifcrf.gov/home.jsp).

### qRT-PCR

The single-stranded cDNA required in the polymerase chain reaction (PCR) analysis was produced using 500 ng of extracted total RNA as a templates for RT (Superscript III Reverse Transcriptase and random primer, Invitrogen, Renfrew, UK). The RT reaction was performed at 65°C for 5 minutes, followed by 25°C for 5 minutes, 50°C for 1 hour, and 70°C for 15 minutes to inactivate the activity of the reverse transcriptase. The synthesized cDNA was diluted 10∶1 in distilled water and used as a template for qRT-PCR, which was performed using the ABI 7300 RT-PCR system (Applied Biosystems, Warrington, UK). Samples of 25 µl containing 1× Universal TaqMan Master Mix (4369016, Applied Biosystems), PCR primers at a concentration of 0.9 µM, and the diluted cDNA were prepared in triplicate. The amplification conditions were 50°C for 2 minutes and 95°C for 10 minutes, followed by 40 cycles of 95°C for 15 seconds and 60°C for 1 minute. The following TaqMan gene expression assay primers (Applied Biosystems) were used: chemokine (C-C motif) ligand 21 (*CCL21*), laminin subunit γ-2 (*LAMC2*), insulin-like growth factor 2 mRNA-binding protein 1 (*IGF2BP1*), interleukin (IL)-6 (*IL6*), ADAM metalloproteinase with thrombospondin motifs-like 3 (*ADAMTSL3*), and leucine-rich repeat transmembrane neuronal protein 1 (*LRRTM1*). 18S rRNA which is known to be a reliable housekeeping gene, was used as an internal control [21]–[23]. ABI 7300 SDS 1.3.1 software (Applied Biosystems) recorded the fluorescence intensity of the reporter and quencher dyes; the results are plotted versus time, quantified as the cycle number. A precise quantification of the initial target was obtained by examining the amplification plots during the early log phase of product accumulation above background [the threshold cycle (Ct) number]. Ct values were subsequently used to determine ΔCt values (ΔCt = Ct of the gene minus Ct of the 18S rRNA control), and differences in Ct values were used to quantify the relative amount of PCR product, expressed as the relative change by applying the equation 2^−ΔCt^. The specific primer assay ID and product sizes for each gene are listed in Table 1.

**Table 1:** Quantitative RT-PCR primers used in this study.

Genes	Primer Assay ID	Product Size (bp)
*LAMC2*	Hs01043711_m1	79
*CCL21*	Hs00171076_m1	81
*IGF2BP1*	Hs00198023_m1	69
*IL6*	Hs00985639_m1	66
*ADAMTSL3*	Hs00324954_m1	91
*LRRTM1*	Hs00611303_m1	101
*18S rRNA*	Hs03003631_g1	69

### IHC staining

IHC staining was performed in order to determine where the genes were expressed. In preparation for IHC staining, deciduous and permanent teeth were fixed in 10% buffered formalin (Sigma, St. Louis, MO, USA) for 1 day, decalcified with 10% EDTA (pH 7.4; Fisher Scientific, Houston, TX, USA) for 8 weeks, embedded in paraffin, and then sectioned at a thickness of 3 µm. The sections were subjected to IHC staining with antihuman IGF2BP1 (rabbit polyclonal, diluted 1∶100; Ab82968, Abcam, Cambridge, UK), antihuman IL6 (rabbit polyclonal, diluted 1∶1600; Ab6672, Abcam), antihuman LRRTM1 (rabbit polyclonal, diluted 1∶50; Ab102968, Abcam), and antihuman ADAMTSL3 (rabbit polyclonal, diluted 1∶50; NBP1-81426, Novus Biologicals, CO, USA). Endogenous peroxidase activity was quenched with 3% hydrogen peroxide. The sections were then incubated in 5% bovine serum albumin (Sigma) to block nonspecific binding and then incubated overnight with primary antibodies, which had been diluted to give optimal staining. After incubation, EnVision+ System-HRP-labeled polymer anti-rabbit (K4003, Dako North America, Carpinteria, CA, USA; ready to use) was applied for 20 minutes or Vectastain Elite ABC Kit (PK-6105, Vector Laboratories, Burlingame, CA, USA; goat IgG, diluted 1∶200) was applied for 30 minutes. Labeled streptavidin biotin kits (Dako) were used for color development according to the manufacturer's instructions. The sections were counterstained with Gill's hematoxylin (Sigma). Control sections were treated in the same manner but without primary antibodies.

## Results

### Gene expression profiles of deciduous and permanent PDL tissues

The distribution and frequency of all of the data were first confirmed using density and box plots. The cDNA microarray results, presented as plots of the standardized log intensity ratio (M) to the average intensity (A) in three deciduous PDL tissues and three permanent PDL tissues (i.e., “M-A” plots), are shown in Figure S1. M-A plots are used to visualize intensity-dependent ratios of raw microarray data, and provide an easy-to-understand overview of the data distribution. These plots confirmed the normalization and standardization of the distributions of the data yielded in this study.

The results demonstrate that the expressions of 51 genes were up-regulated by twofold or more in one tissue type relative to the other. Seven genes (five in deciduous PDL tissue and two in permanent PDL tissue) were not identified well. Ultimately, 44 genes were analyzed further. In deciduous PDL tissues, the expressions of 16 genes were up-regulated by twofold or more compared to permanent PDL tissue, especially *LAMC2*, cartilage oligomeric matrix protein (*COMP*), and *CCL21* were expressed more than threefold (Table 2). Whereas, those of 28 genes were up-regulated by twofold in permanent compared to deciduous PDL tissue, especially *IL6*, SH3 and cysteine-rich domain (*STAC*), and FBJ murine osteosarcoma viral oncogene homolog B (*FOSB*) were found to be up-regulated by more than fourfold (Table 3).

**Table 2:** Up-regulated genes in deciduous PDL tissue (compared to permanent PDL tissue).

Gene Description	Gene Symbol	Fold Change	Gene Accession	Cytoband
laminin, gamma 2	*LAMC2*	3.25	NM_005562	1q25-q31
cartilage oligomeric matrix protein	*COMP*	3.19	NM_000095	19p13.1
chemokine (C-C motif) ligand 21	*CCL21*	3.13	NM_002989	9p13
peptidase domain containing associated with muscle regeneration 1	*PAMR1*	2.76	NM_015430	11p13
chemokine (C-C motif) ligand 18	*CCL18*	2.67	NM_002988	17q11.2
coagulation factor II (thrombin) receptor-like 2	*F2RL2*	2.57	NM_004101	5q13
interleukin 1, alpha	*IL1A*	2.53	NM_000575	2q14
insulin-like growth factor 2 mRNA binding protein 1	*IGF2BP1*	2.48	NM_006546	17q21.32
Fc fragment of IgG, low affinity IIIa, receptor (CD16a)	*FCGR3A*	2.47	NM_000569	1q23
laminin, beta 3	*LAMB3*	2.43	NM_001017402	1q32
phosphate-regulating endopeptidase homolog, X-linked	*PHEX*	2.19	NM_000444	Xp22.2-p22.1
mab-21-like 2 *(C. elegans)*	*MAB21L2*	2.16	NM_006439	4q31
small proline-rich protein 2B	*SPRR2B*	2.10	NM_001017418	1q21-q22
paired box 3	*PAX3*	2.10	NM_181458	2q35-q37|2q35
tetraspanin 2	*TSPAN2*	2.06	NM_005725	1p13.2
immunoglobulin kappa variable 1–5	*IGKV1-5*	2.05	ENST00000453166	2p12

**Table 3:** Up-regulated genes in permanent PDL tissue (compared to deciduous PDL tissue).

Gene Description	Gene Symbol	Fold Change	Gene Accession	Cytoband
interleukin 6 (interferon, beta 2)	*IL6*	4.95	NM_000600	7p21
SH3 and cysteine rich domain	*STAC*	4.19	NM_003149	3p22.3
FBJ murine osteosarcoma viral oncogene homolog B	*FOSB*	4.02	NM_006732	19q13.32
cellular retinoic acid binding protein 1	*CRABP1*	3.74	NM_004378	15q24
ADAMTS-like 3	*ADAMTSL3*	3.51	NM_207517	15q25.2
FBJ murine osteosarcoma viral oncogene homolog	*FOS*	2.97	NM_005252	14q24.3
dedicator of cytokinesis 3	*DOCK3*	2.91	NM_004947	3p21.2
phosphodiesterase 3B, cGMP-inhibited	*PDE3B*	2.81	NM_000922	11p15.1
leucine rich repeat transmembrane neuronal 1	*LRRTM1*	2.71	NM_178839	2p12
ADAM metallopeptidase with thrombospondin type 1 motif, 18	*ADAMTS18*	2.64	NM_199355	16q23
corin, serine peptidase	*CORIN*	2.57	NM_006587	4p13-p12
cAMP-regulated phosphoprotein, 21 kDa	*ARPP21*	2.55	NM_016300	3p22.3
hypothetical LOC644714	*LOC644714*	2.50	BC047037	3p21.31
V-kit Hardy-Zuckerman 4 feline sarcoma viral oncogene homolog	*KIT*	2.33	NM_000222	4q11-q12
neural cell adhesion molecule 2	*NCAM2*	2.29	NM_004540	21q21.1
sestrin 3	*SESN3*	2.27	NM_144665	11q21
leucine rich repeat transmembrane neuronal 3	*LRRTM3*	2.26	NM_178011	10q21.3
protease, serine, 12 (neurotrypsin, motopsin)	*PRSS12*	2.24	NM_003619	4q28.1
calsequestrin 2 (cardiac muscle)	*CASQ2*	2.21	NM_001232	1p13.3-p11
myosin, heavy chain 10, non-muscle	*MYH10*	2.17	NM_005964	17p13
ribosomal protein L21	*RPL21*	2.16	NM_000982	13q12.2
secreted frizzled-related protein 1	*SFRP1*	2.13	NM_003012	8p11.21
synaptotagmin I	*SYT1*	2.12	NM_005639	12cen-q21
solute carrier family 22, member 3	*SLC22A3*	2.12	NM_021977	6q25.3
cytochrome P450, family 1, subfamily B, polypeptide 1	*CYP1B1*	2.11	NM_000104	2p21
jun proto-oncogene	*JUN*	2.08	NM_002228	1p32-p31
small nucleolar RNA, H/ACA box 4	*SNORA4*	2.07	NR_002588	3q27
adenylate cyclase 10 pseudogene	*LOC221442*	2.05	NR_026938	6p21.1

### GO analysis

**Figure 1:**
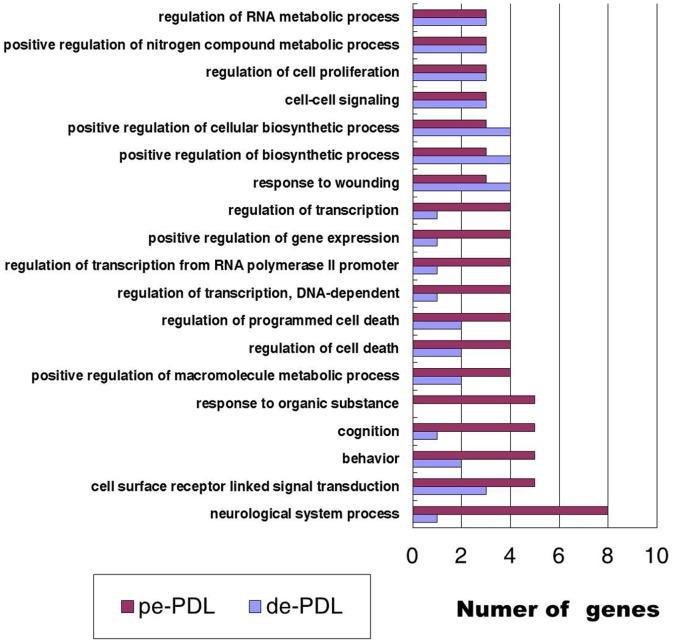
Main categories of genes expressed specifically in deciduous PDL tissues (de-PDL) and permanent PDL tissues (pe-PDL) on the basis of their biological processes. Numbers of involved genes are listed in *x*-axis, F-statistic *p*<0.05.

GO classes with an F-statistic *p* value of <0.05 following analysis on the basis of their biological processes are shown in Figure 1. Those GO-class processes found more frequently in the PDL tissues of permanent teeth include neurological system processes, cognition, and response to organic substances. In contrast, the GO-class processes found more frequently in the PDL tissues of deciduous teeth included positive regulation of cellular biosynthetic processes and response to wounding.

**Figure 2:**
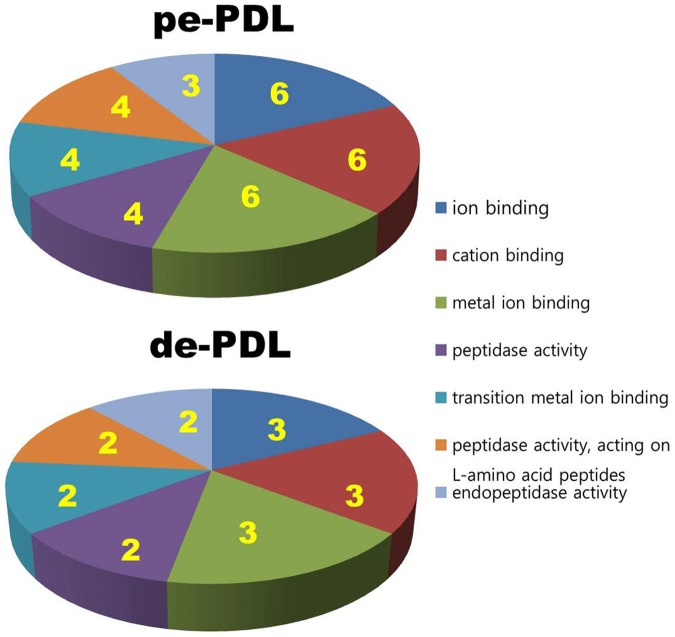
Main categories of genes expressed specifically in de-PDL and pe-PDL on the basis of their molecular functions. Numbers of involved genes are listed in the pie charts, F-statistic *p*<0.05.

The GO classes with an F-statistic *p* value of <0.05 following analysis on the basis of their molecular functions are shown in Figure 2. These classes did not differ noticeably between the PDL tissues of deciduous teeth and those of permanent teeth.

### qRT-PCR

qRT-PCR analysis was performed to verify the differential expression levels determined via cDNA microarray analysis. Among interesting genes, we selected three genes that are expressed more in the deciduous PDL tissues and three genes that are expressed more in the permanent PDL tissues. The six genes (i.e., *LAMC2*, *CCL21*, *IGF2BP1*, *IL6*, *LRRTM1*, and *ADAMTSL3*) that were selected for this verification procedure exhibited an increase of at least fourfold in the gene expression level compared to the other tissue types (Table 4). The results are consistent with those of the cDNA microarray analysis.

**Table 4:** The relative gene expressions in the deciduous and permanent PDL tissues.

Gene	Relative Gene Expression (mean±SE)	
	Deciduous PDL Tissues	Permanent PDL Tissues
*LAMC2*	4.90±0.20	1
*CCL21*	6.02±0.03	1
*IGF2BP1*	43.78±0.00	1
*IL6*	1	34.38±0.02
*ADAMTSL3*	1	4.02±0.06
*LRRTM1*	1	12.67±0.05

SE; standard error.

### IHC staining

**Figure 3:**
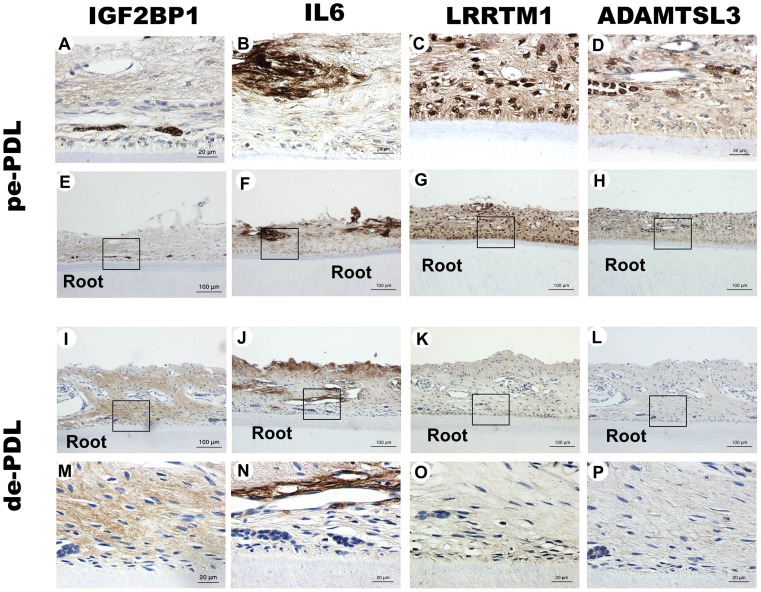
Immunohistochemical (IHC) staining of permanent and deciduous PDL tissues (A–P). (**A**, **E**) IHC staining for IGF2BP1 in permanent and (**I**, **M**) deciduous PDL tissues. (**B**, **F**) IHC staining for IL6 in permanent and (**J**, **N**) deciduous PDL tissues. (**C**, **G**) IHC staining for LRRTM1 in permanent and (**K**, **O**) deciduous PDL tissues. (**D**, **H**) IHC staining for ADAMTSL3 in permanent PDL tissues and (**L**, **P**) deciduous PDL tissues. Abbreviations: pe-PDL, permanent PDL tissues; de-PDL, deciduous PDL tissues. Scale bars: 100 µm in **A–D** and **M–P**; 20 µm in **E–H** and **I–L**.

The following four proteins were the targets of the IHC study: IGF2BP1, IL6, ADAMTLS3, and LRRTM1 (Figure 3). IGF2BP1 was broadly stained in the deciduous PDL tissues, but only weakly in permanent PDL tissues, with the exception of the epithelial cell rests of Malassez. ADAMTLS3 and LRRTM1 were stained more strongly across the board in permanent compared to deciduous PDL tissues. IL6 was located mainly around blood vessels, and its staining was more prominent in the permanent PDL tissues.

## Discussion

The periodontal tissues used in this study were located at the surface of the extracted teeth. PDLs usually tear across their middle during tooth extraction, and they contain not only PDL fibroblasts, epithelial cell rests of Malassez, cementoblasts, vascular cells, and sensory nerve cells, but also osteoblasts, which make and maintain the alveolar bone. However, since there are no reports about differences in cell populations between the PDLs of permanent and deciduous teeth, the obtained RNAs must have originated from these cell populations.

Just 51 of the known >20,000 human genes [24] were differentially expressed by more than twofold between the two PDL tissue types. This indicates that while differences do exist between the tissues, their compositions and functions are very similar. There were similar quantities of genes predominantly or specific expressed in PDLs (periostin, scleraxis, and collagen XII) [25]–[27] and cementoblasts [cementum protein-23 (*CP-23*) and cementum attachment protein (*CAP*)] [28], [29]. In addition, inhibitors of PDL mineralization (aspirin) [30], matrix metalloproteinases (MMPs) [31], which are involved with turnover of the PDL matrix, and factors associated with osteoclast differentiation [*RANKL* and osteoprotegerin (*OPG*)] [32] were expressed to similar degrees in the two tissue types. This similarity probably explains why histological differences between permanent PDLs and deciduous PDLs have seldom been reported previously.

The changes that the PDL undergoes during the root resorption of deciduous teeth have been investigated previously. Fukushima et al. [12] insisted that cells obtained from the root-resorbing area of deciduous PDLs expressed more RANKL (encoding a factor that enhances osteoclastogenesis) and less OPG (encoding a factor that suppresses osteoclastogenesis), and that cells isolated from either nonresorbing deciduous teeth or permanent teeth expressed equal ratios of RANKL and OPG, in line with our present findings. Furthermore, one group reported that the mucopolysaccharidase and collagenolytic activities of deciduous PDL were higher in a resorbing root than in nonresorbing roots and permanent PDLs [17], [33]. Another study showed that the IHC signals of bone sialoprotein and osteopontin, which contain the osteoclast-binding RGD sequence, were higher in the root-resorbing area than in normal deciduous PDL and permanent PDL areas [10]. Our finding of no differences in those genes between the two types of PDL tissues is probably due to the PDL in the root-resorbing area not being included in the analysis. However, although several of the molecular and cellular events that occur during the resorption of deciduous roots have been revealed, further investigation is required to determine which factor awakens silent deciduous PDLs to become involved in the root-resorbing process.

Several of the genes that were up-regulated in deciduous PDL tissues were associated with the formation of the extracellular matrix and organ development. For example, *LAMC2* and laminin beta 3 (*LAMB3*) encode the laminin family proteins, which are components of the basement membrane. Laminin is known to be involved in a wide variety of biological processes such as cell adhesion, differentiation, migration, signaling, neurite outgrowth, and metastasis [34], while cartilage oligomeric matrix protein (*COMP*) is known to be a type of noncollagenous extracellular matrix protein, a marker of cartilage turnover [35], and an abundant component of tendon [36]. *IGF2BP1* plays an important role in control dental development [37], and regulates the growth factor IGF2. Phosphate-regulating endopeptidase homolog, X-linked (*PHEX*) is involved in bone and dentin mineralization and renal phosphate reabsorption [38]. It is thought that Mab-21-like 2 (*C. elegans*) (*MAB21L2*) is involved in neural development [39]. Paired box 3 (*PAX3*) has been identified in association with fetal development [40]. The present study is the first to demonstrate that these genes are expressed more strongly in deciduous PDL than permanent PDL, but their precise functions in periodontal tissues remain to be elucidated.

Interestingly, in spite of their origin in the nonresorbed area, the deciduous PDL tissues expressed more genes associated with inflammation or immune reaction than did the permanent PDL tissues. The biological function of CCL21 and chemokine (C-C motif) ligand 18 (CCL18) is chemotaxis for lymphocytes, the inflammatory response, and the immune response [41], [42]. In addition, these chemoattractants exhibit a slight chemotactic activity for monocytes [43], [44], which are precursors of osteoclasts or odontoclasts. It is therefore suggested that these chemokines are involved in the degradation of deciduous PDL tissues and roots. Interleukin 1, alpha (IL1A) is involved in the immune response, inflammatory processes, and apoptosis [45], [46], and can enhance the biosynthesis of prostaglandin [47] and some kinds of MMPs [48]. In addition, it up-regulates *RANKL* and down-regulates *OPG* in PDL cells [49], and positively affects the survival and differentiation of osteoclasts or odontoclasts and consecutive bone or tooth resorption [50]–[52]. Therefore, deciduous PDL tissues appear to be prone to resorption because of the relatively strong expressions of genes associated with tissue destruction even in the normal functioning state.

Several of the up-regulated genes in permanent PDL tissues seem to be associated with degradation of the extracellular matrix. *IL6* functions as a proinflammatory cytokine and plays a role in orthodontic movement and lipopolysaccharide-induced inflammatory reactions [53]–[55]. *ADAMTS18* belongs to the ADAMTS family of extracellular proteases, which are zinc-dependent metalloproteinases that play an important role in both normal and pathological events, and especially in connective tissue organization, coagulation, inflammation, arthritis, angiogenesis, and cell migration [56]. It has been reported that PDL cells and cementoblasts regulate the extracellular accumulation of a large extracellular matrix proteoglycan using the degrading enzymes *ADAMTS1*, *-4*, and *-5*, which are members of ADAMTS family [57]. Interestingly, stimulation of *IL6* up-regulates the expressions of *ADAMTS4* and *ADAMTS-5* [58]. Although it is unknown whether *IL6* and *ADAMTS18* are related, genes that were shown in the present study to be up-regulated in permanent PDL tissues are likely to play a part in the turnover of the extracellular matrix in permanent PDL tissues.

On the other hand, several of the genes that are relatively strongly expressed in permanent PDL tissues are associated with myocontraction. In the present study, IL6 was found mainly around the blood vessels, and it is known that *IL6* induces the expression of vascular endothelial growth factor and is associated with angiogenesis [59]. Phosphodiesterase 3B, cGMP-inhibited (*PDE3B*) is expressed in vascular smooth muscle cells and is thought to modulate contraction [60], [61]. Calsequestrin 2 (*CASQ2*) is a calcium-binding protein of the sarcoplasmic reticulum in cardiac and slow skeletal muscle; the release of calsequestrin-bound calcium triggers muscle contraction [62]. Myosin, heavy chain 10, non-muscle (*MYH10*) regulates cell adhesion and migration [63]. While it is not certain that these aforementioned genes are related to the presence of abundant blood vessels or abundant myofibroblasts, which are generally present in organs with a high remodeling capacity such as the PDL [64], unfortunately there are no reports of quantitative differences in blood vessels or myofibroblasts between permanent and deciduous PDL tissues.

Some of the genes that were relatively strongly expressed in permanent PDL tissues are associated with neural function. According to the GO groupings identified for biological processes and molecular functions, the genes involved in neurological system processes were expressed much more strongly in permanent than in deciduous PDL tissues. Only *PAX3* plays a role in this function in deciduous PDL tissues, but in permanent PDL tissues, eight genes were identified: *FOS*, V-kit Hardy-Zuckerman 4 feline sarcoma viral oncogene homolog (*KIT*), neural cell adhesion molecule 2 (*NCAM2*), *MYH10*, synaptotagmin I (*SYT1*), solute carrier family 22, member 3 (*SLC22A3*), cytochrome P450, family 1, subfamily B, polypeptide 1 (*CYP1B1*), and jun proto-oncogene (*JUN*). Among these genes, *FOS* is known to be an indirect marker of neuronal activity because it is often expressed when neurons fire action potentials [65], *NCAM2* is a type I membrane protein and is thought to induce neurite outgrowth [66], [67], *SYT1* functions as a calcium regulator of neurotransmitter release [68], and *SLC22A3* is an extraneuronal monoamine transporter and is involved in neurotransmission [69]. Furthermore, among the genes that are relatively strongly expressed in permanent PDL tissues, dedicator of cytokinesis 3 (*DOCK3*), *LRRTM1*, leucine-rich repeat transmembrane neuronal 3 (*LRRTM3*), protease, serine 12 (*PRSS12*), and cAMP-regulated phosphoprotein, 21 kDa (*ARPP21*) are genes known to be associated with neuronal signaling systems. *DOCK3* is specifically expressed in neurons, especially in the central nervous system, and induces axonal outgrowth [70]. *LRRTM1* and *LRRTM3* are transmembrane proteins that contain many leucine-rich repeats; they are found in neurons and are associated with synaptic function [71], [72]. *PRSS12* is a mosaic serine protease that is expressed preferentially in motor neurons, and defects of this gene are correlated with mental retardation [73]. *ARPP21* is enriched in the caudate nucleus and cerebellar cortex, and regulates the effects of dopamine [74]. Although we were unable to determine the precise function of these genes, the up-regulation of genes related to neurological system processes in permanent PDL tissues is consistent with the degree of innervation being lower for deciduous incisors and canines than for permanent premolars [3], [75], [76].

In conclusion, the genes in deciduous PDL tissues that are up-regulated relative to their permanent counterparts are involved in the formation of the extracellular matrix and inflammation or immune reactions, leading to PDL degradation and root resorption. Those that are up-regulated in permanent compared to deciduous PDL tissues are related to the regulation of extracellular components, myocontraction, and neurological responses. Although only the RNA from the entire periodontal tissue of permanent and deciduous teeth was investigated in this study, and not from the individual diverse types of cells that constitute these tissues, our results provide clues as to their respective functions and support the histologic findings for permanent and deciduous PDL tissues.

## Supporting Information

Figure S1 **M-A plot comparing three deciduous PDL tissue samples and three permanent PDL tissue samples.** In each plot the *x*-axis (A) is 0.5×(log_2_(case)+log_2_(control)) and the *y*-axis (M) is log_2_(case/control). M and A correspond to the difference between the log intensities and the average log intensity, respectively. The data of all plots were normally distributed.
(TIF)
