# Supplementary figures and images for: Comparative Gene Expression Analysis of the Human Periodontal Ligament in Deciduous and Permanent Teeth
Source: PLoS One. 2013 Apr 8;8(4):e61231. doi: 10.1371/journal.pone.0061231 (PMC3620385; doi:10.1371/journal.pone.0061231)

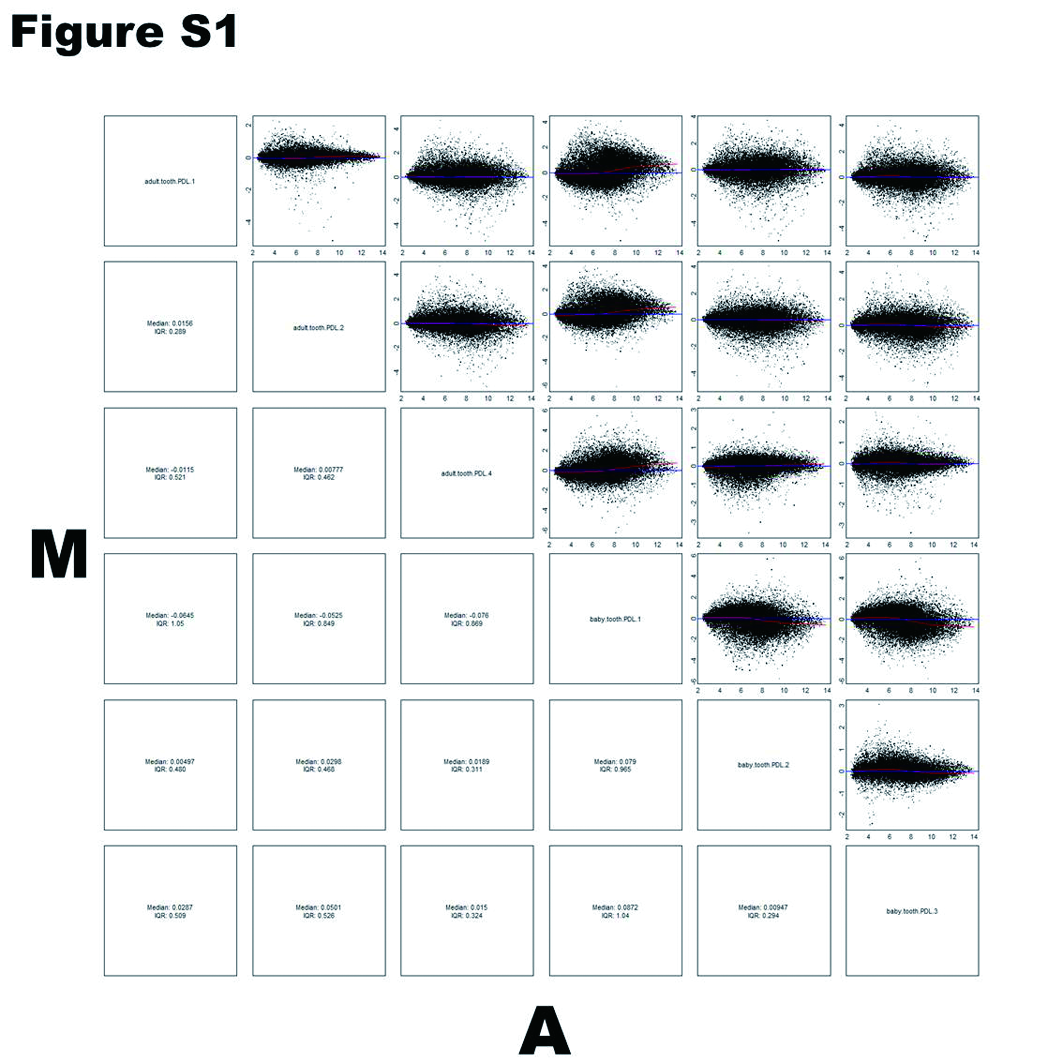

Supplement: Figure S1 — M-A plot comparing three deciduous PDL tissue samples and three permanent PDL tissue samples. In each plot the x-axis (A) is 0.5×(log2(case)+log2(control)) and the y-axis (M) is log2(case/control). M and A correspond to the difference between the log intensities and the average log intensity, respectively. The data of all plots were normally distributed. (TIF) [file pone.0061231.s001.tif]
